# SD-OCT Biomarkers and the Current Status of Artificial Intelligence in Predicting Progression from Intermediate to Advanced AMD

**DOI:** 10.3390/life12030454

**Published:** 2022-03-19

**Authors:** Ioana Damian, Simona Delia Nicoară

**Affiliations:** 1 Department of Ophthalmology, “Iuliu Hatieganu” University of Medicine and Pharmacy, 8 Victor Babeș Street, 400012 Cluj-Napoca, Romania; ioana.damian@umfcluj.ro; 2 Clinic of Ophthalmology, Emergency County Hospital, 3-5 Clinicilor Street, 40006 Cluj-Napoca, Romania

**Keywords:** intermediate age-related macular degeneration progression, SD-OCT, artificial intelligence, imaging biomarkers, deep learning

## Abstract

Age-related macular degeneration (AMD) is one of the leading causes of blindness in the Western World. Optical coherence tomography (OCT) has revolutionized the diagnosis and follow-up of AMD patients. This review focuses on SD-OCT imaging biomarkers which were identified as predictors for progression in intermediate AMD to late AMD, either geographic atrophy (GA) or choroidal neovascularization (CNV). Structural OCT remains the most compelling modality to study AMD features related to the progression such as drusen characteristics, hyperreflective foci (HRF), reticular pseudo-drusen (RPD), sub-RPE hyper-reflective columns and their impact on retinal layers. Further on, we reviewed articles that attempted to integrate biomarkers that have already proven their involvement in intermediate AMD progression, in their models of artificial intelligence (AI). By combining structural biomarkers with genetic risk and lifestyle the predictive ability becomes more accurate.

## 1. Introduction

AMD is a progressive degenerative disease of the retina and one of the leading causes of blindness in the Western World. The projections have shown that 196 million people will have AMD in 2020, while for 2040, 288 million people are expected to develop the disease [1], of which 18.6 million the advanced form [2]. Its prevalence is continuously growing due to the ageing of the population. A systematic review from 2014 has shown a pooled prevalence of 8.7% for the age range of 45–85 years [1]. Since the irreversible central vision loss due to AMD is highly incapacitating, patients experience emotional, physical but also social issues. A strong impact is felt by the health system since the management of this disease requires consistent human and financial resources. 

Histologically and clinically two subtypes of AMD were described: atrophic AMD (aAMD) and neovascular (nvAMD). aAMD progresses slowly, and it manifests clinically as drusen, focal or geographic atrophy (GA) of retinal pigment epithelium (RPE) and photoreceptors’ disfunction followed by their degeneration. On the other side, nvAMD is more debilitating. The hallmark of nvAMD are the new vessels, that grow either into the sub-RPE space, the sub-retinal space or intraretinally as in retinal angiomatous proliferation [3].

Depending on drusen features and pigment abnormalities, the following classification is currently used for AMD. (See Table 1).

Traditionally, the clinical staging of AMD is performed using either ophthalmoscopy or color fundus photography (CFP). The emergence of OCT enabled clinicians and researchers to characterize the microstructural alterations in different layers of the retina [4]. Due to the ease of acquisition and ubiquity of OCT, this imaging method has become an attractive option for studying biomarkers involved in the diagnosis, prognosis, and progression of AMD. Bearelly et al. and Brar et al. suggested that OCT may be useful in monitoring the progression to GA, since it is capable to measure photoreceptor loss at GA margins which also correlates with the fundus autofluorescence changes [5,6]. However, since the scan analysis is prone to human error as well as variability, major steps were made in developing automated algorithms able to recognize the early signs of AMD as well as to assess its severity and predict progression. 

Since one of the major challenges in the management of patients with intermediate AMD is to assess the risk of conversion to the advanced stage, motivated by this need, we conducted a review with the purpose to provide an overview of the current literature investigating OCT imaging biomarkers predictive for progression which are detectable in intermediate AMD. Furthermore, we searched for articles that integrated some of these biomarkers in artificial intelligence (AI) models.

## 2. Method of Literature Search

### 2.1. Study Selection

A comprehensive review of literature was performed through a PubMed search in November 2021. We used the following keywords in various combinations: intermediate age-related macular degeneration, progression, predictive, optical coherence tomography, imaging biomarkers, artificial intelligence, deep learning. When a specific imaging biomarker was identified, we also used it as a keyword in a subsequent PubMed search to identify additional publications. Articles cited in the reference list were also reviewed whenever they were considered relevant. Initially, titles and abstracts were reviewed for eligibility. All eligible, full-text publications were examined for inclusion and exclusion criteria. 

The inclusion criteria for papers regarding SD-OCT biomarkers were as follows: (1) studies reporting SD-OCT biomarkers in intermediate AMD; (2). studies reporting progression from intermediate to advanced AMD; (3). English-language literature only. The exclusion criteria were as follows: (1). unpublished studies; (2). publications including comments, letters, editorials, reviews, case reports; (3). studies applying only retinal photography, fundus fluorescein angiography (FFA), OCTA or genetic risk factors for detecting prognostic factors.

The inclusion criteria for papers regarding AI involvement in predicting AMD progression were as follows: (1). studies reporting predictive SD-OCT biomarkers using AI-based algorithm; (2). studies providing clear information about the database and number of images in various data sets; (3). studies providing information on evaluation indices such as area under the curve (AUC); (4). English-language literature only. The exclusion criteria were as follows: (1). unpublished studies; (2). studies applying retinal photography for detecting prognostic factors; (3). publication including comments, letters, editorials, reviews. 

### 2.2. Quality Assessment of Included Studies

The quality of the papers was appraised by assessing the STARD (Standard of reporting for diagnostic accuracy studies) reporting guidelines for diagnostic and prognostic (see Table S1). The quality of AI papers was appraised by assessing the CONSORT-AI (Consolidated Standards of Reporting Trials-Artificial Intelligence) reporting guidelines (see Table S2). 

## 3. Degeneration of RPE in AMD’s Pathogenesis

Regarding AMD’s pathogenesis, two hypotheses have emerged: the first assumption endorses that RPE’s atrophy consequently induces choriocapillaris loss and photoreceptors’ degeneration [3]. By contrast, the second theory states that choroidal vascular insufficiency secondarily produces RPE’s dysfunction and photoreceptors’ degeneration [3]. Nevertheless, the relationship between RPE and choriocapillaris is mutual. If one of them is compromised, either one or both become dysfunctional or even degenerate. McLeod et al. have demonstrated that a selective damage of RPE with sodium iodate or by mechanical debridement, induces choriocapillaris atrophy [3]. In addition, these changes are not limited to choriocapillaris perfusion, but they seem to influence the flow within the large choroidal blood vessels [7]. After RPE is removed, changes in choriocapillaris perfusion are due to the loss of RPE-derived trophic factors, responsible for maintaining the endothelial integrity which secondarily induces choriocapillaris atrophy [8]. The factors involved in choroidal endothelial cells survival are basic fibroblast growth factor (bFGF), vascular endothelial growth factor (VEGF), endothelin-1 and insoluble molecules from the extracellular matrix produced by RPE [2]. VEGF produced by RPE stimulates the formation of fenestrations, induces the angiogenesis and it is also known as an endothelial cell survival factor [3]. Studies in vitro have revealed that RPE secretes VEGF basally, towards the choriocapillaris [9]. In humans, VEGF receptors are expressed on the choroidal endothelium that faces RPE [9]. Consistent with these results, McLeod et al. concluded that in GA, because RPE is atrophied, the source of VEGF is removed, which will further lead to the loss of capillaries’ fenestration. [3]. 

Nevertheless, the mechanism behind RPE’s degeneration in AMD is multifactorial: environmental factors, intense light exposure, oxidative stress, and genetics are all being involved. The genetic factor was demonstrated by numerous studies that highlighted 52 common variants which are associated with AMD among another 100 rare variants [10]. A significant proportion of AMD causes where explained by these variants which also brought insights regarding involved pathogenesis pathways such as complement pathway, one of the most important. As shown by Colijn et al., in Europe the risk allele most discriminative between late AMD and control is localized in ARMS2, closely followed by a risk-increasing and protective allele in CFH [2]. Additionally, in late AMD patients, a positive total genetic risk score (GRS) of 90% was identified in 1581 of 1777 patients (89%). The ARMS2 and complement pathway proved to be the most prominent genetic pathways in late AMD (90% of the patients) [2]. The same researchers found that rs3750846 (or its proxy, rs10490924, A69S) variant in the ARMS2 locus carried the highest risk of late AMD and the second highest attribution to overall AMD occurrence in their study group [2]. Lifestyle was also identified as a strong determinant of the outcome in each genetic risk category: unfavorable lifestyle described as a lower intake of vegetables, fish, fruit and higher rate of smoking increased the risk of late AMD at least 2-fold [11]. 

## 4. Predictive OCT Biomarkers

OCT is known as an “in vivo” imaging method capable of resolving cross-sectional retinal substructures [12]. Spectral domain (SD-OCT) or swept-source (SS-OCT) has become the backbone for the diagnosis, follow-up, and treatment evaluation in AMD patients. Previous studies indicated it is potentially valuable in providing imaging features that could be used to predict AMD progression. All SD-OCT images that appear in the review illustrating the OCT biomarkers were acquired using a Spectralis OCT (Heidelberg Engineering, Inc., Heidelberg, Germany).

### 4.1. Druse Qualitative and Quantitative Features

Druse is a key pathognomonic feature for AMD. It is one of the first signs in AMD, being a hallmark of this disease. On clinical examination it appears as a yellowish deposit in the macular area. Druse is characterized as hard or soft and regarding the size it is either small (<63 µm), intermediate (>63 µm but <125 µm) or large (≥125 µm) [13]. Traditionally, druse has been assessed during ophthalmoscopy examination or on CFP. Since the emergence of SD-OCT, its analysis became more complex, we are now able to follow features such as height, diameter, volume, area, shape, internal reflectivity, homogeneity and its relationship with the RPE [14]. SD-OCT technology offers the chance to perform a cross-sectional and volumetric analysis of high resolution. On OCT scan drusen appear as RPE’s elevations of different sizes and shapes, while the sub-RPE material has a medium or high reflectivity. 

Up to 47% of the total drusen are homogenous with a medium internal reflectivity and with no overlying foci [14]. The remaining drusen have different features, one study has identified 17 different patterns after analyzing 120 drusen [15]. Their distinctive appereance seems to be explained by the different proportion between lipids and proteins in their composition [16]. 

Analysing the volume of a druse seems to be crucial for the insight into mechanism of the disease. For instance, a druse with a volume greater than 0.03 mm^3^ situated in the central 3 mm-diameter circle of the macula, occurring in up to 25 per cent of intermediate AMD eyes, has been connected to a four-fold higher risk of progression to advanced AMD when the fellow eye was treated for nvAMD [17]. 

Drusen are dinamic structures that evolve and regress in time, thus providing information regarding the risk of disease progression. OCT technology has made it possible to visualize subtle morphological features. Several studies have enhanced that drusen suffer repeated volume growing cycles in a cubic pattern compared to regression that takes place very fast [13,18]. Druse’s regression seems to be dependent of its initial volume: larger the druse volume, a higher tendency of regression was found (*p* = 0.001, 3 mm circle) [13]. Other studies identified the size of the druse as a risk factor for progressing to advanced AMD. An increased height compresses or even disrupts the ellipsoid zone (EZ), external limiting membrane (ELM) and outer nuclear layer (ONL) which translates into a negative outcome [19]. A larger volume is responsible for an increased probability for spontaneous regression which could be construed into a higher risk of progression to GA or CNV [13]. Following drusen regression, the more common outcome seems to be GA rather than CNV [19]. Furthermore, over 50% of drusen from intermediate AMD eyes present fading or regression over a two-year period [19]. In terms of clinical changes, 96% of the eyes that went to develop GA had drusen larger than 125 µm, 83% drusen larger than 250 µm, 94% had confluent drusen, 96% focal hyperpigmentation and in 82% drusen experienced regression and hypopigmentary changes, as reported by AREDS research group [20]. Average time from baseline to initial appearance of GA was 6.6 years, which also varied by lesion type ranging from 2.5 years for hypopigmentation and 5.9 years for drusen confluence [20]. 

**Figure 1:**
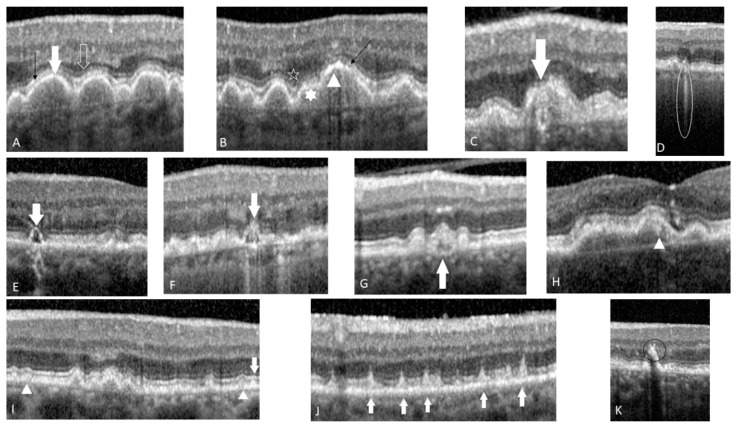
Predictive OCT biomarkers: (**A**) the white full arrow indicates continuous RPE, the black arrow indicates continuous EZ and the white empty arrow a continuous ELM; (**B**) the white arrow head indicates thicker RPE, and the white full star indicates thinner RPE; the empty star indicates discontinuous ELM and the black arrow discontinuous EZ; (**C**) the t white arrow indicates interrupted RPE, EZ and ELM; (**D**) the white circle indicates a thin sub-RPE hyper-reflective column; (**E**) L-type ODS: the white arrow indicates a focal, well-circumscribed sub volume of distinct hypo-reflectivity within the druse. (**F**) C-type ODS: druse with a conical shape indicated by the white arrow; (**G**) S-type ODS: the white arrow indicates a druse split into 2 sub volumes (in any proportion) of distinct levels of reflectivity (low and high); (**H**) H-type ODS: the arrow head indicates a focal, well-circumscribed sub volume of distinct hyperreflectivity within the druse; ELM and EZ are interrupted; (**I**) the white arrow indicates stage 1 RPD: hyper-reflective material between RPE and EZ without interfering with the EZ. while the arrow head indicates stage 2 RPD: increased accumulation of hyper-reflective material forming mounds and distortion of the overlying ellipsoid layer; (**J**) the white arrow indicates stage 3 RPD: characteristic conical shape that has punctured through the EZ and ELM; (**K**) The black circle indicates HRF with high, inhomogeneous internal reflectivity overlying the druse.

Druse’s relationship with RPE affects the integrity of the latter: therefore RPE could be thinner, thicker or even interupted (See Figure 1A–C). It seems that especially the druse situated near the central macula induce RPE’s interruptions [14].

### 4.2. OCT-Reflective Drusen Substructures

**Figure 2:**
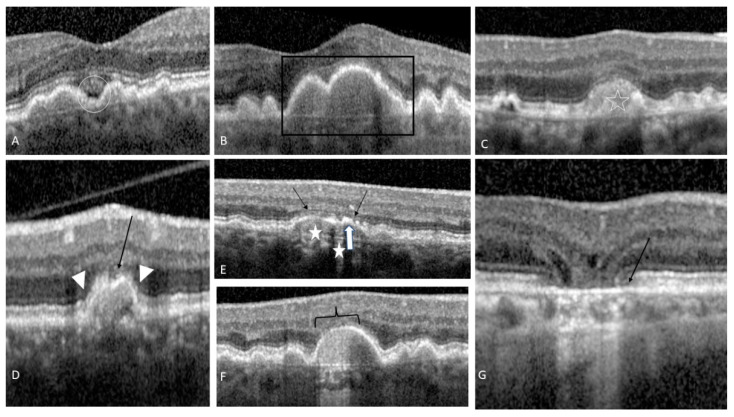
OCT biomarkers for intermediate AMD progression: (**A**) The white circle indicates a hyporeflective space in the concavity between 2 soft drusen, without overpassing their apex; (**B**) the black empty rectangle indicates the PED; (**C**) The white star indicates a well-defined dome-shaped lesion at the level of Bruch membrane and above RPE; (**D**) iORA: thinning of the ONL indicated by the black arrow, loss of ELM indicated by the white arrow head but relatively intact RPE band with no light hyper transmission into the choroid; (**E**) cORA: severe thinning of the ONL indicated by the black arrow, intact RPE band indicated by the white arrow with intermittent hyper transmission of light indicated by the white stars; (**F**) iRORA: degeneration of photoreceptors, an irregular or interrupted RPE band indicated by the curly bracket and discontinued hyper transmission of light; (**G**) cRORA: degeneration of photoreceptors and a zone of complete disrupted RPE band indicated by the black arrow of at least 250 µm in diameter with the hyper transmission of light.

Usually druse appear as a dome-shaped lesion with medium internal homogenous reflectivity that separates RPE from Bruch’s membrane with no overlying foci. The risk for progression results from various aspects, such as shape (pointed, saw-toothed, dome-shaped), reflectivity (low, medium and high) or internal homogeneity (homogeneous, non-homogeneous with or without a central “core”) [19]. The authors of AREDS2 ancillary SD-OCT study termed these appearances ‘optical coherence tomography-reflective drusen substructures’ (ODS) [16]. They differ from hypereflective foci (HRF) by their location under the RPE layer within drusen themselves. They are more transient and reflect the heterogeneity in drusen’s composition and architecture [16]. In intermediate AMD, ODS are relatively rare, 24% of the intermediate AMD eyes present this feature, they do not constitue long-term features and are not preceded by any specific unique lesion [16]. Four subtypes of drusen have been described: H-type (high-reflective core) (see Figure 2H), L-type (low-reflective core) (see Figure 1E), C-type (conical debris) (see Figure 1F) and S-type (split druse) (See Figure 1G). Druse with low or high internal reflectivity compared to the commonly medium one, are more prone to develop an internal core and carry an association with GA [16].

Follow-up data over a two-year period has further revealed a specific association between C-type ODS with new onset GA (3/8, 37.5 per cent vs. 8/163 or 4.91 per cent of eyes with no ODS at baseline; *p* = 0.0094) [16]. Histopathological studies found the presence of spherules within the druse which resemble the ODS. Concentric ring or coiled structures on immunohistochemical analysis with amyloid beta and amyloid precursor protein are localized in the outer shell of the vesicle. More than that, these specific vesicles were found immunopositive for immunoglobulin G, RPE pigment granules and complement components C3b/5/C5b-9 [11,21]. Residual debris from degenerating RPE cells that remain entrapped between the RPE and Bruch’s membrane serve as a chronic inflammatory stimulus for complement attack and a potential site for druse formation [21]. These small spherules were found positive for apolipoprotein E, a common component of the extracellular plaques and deposits characteristic of atherosclerosis, Alzheimer disease, that is upregulated by Müller glia cells in degenerating human retina affected by AMD [22]. Alteration of lipoprotein biosynthesis and processing at the level of RPE and Bruch’s membrane seems to be a contributing factor in drusen formation. 

### 4.3. Reticular Pseudo-Drusen or Subretinal Drusenoid Deposits 

They typically appear as a well-defined round or triangular lesions of hyperreflective material over the RPE in the superior macula or along the supero-temporal vascular arcade. According to a histological study, they are asociated with the loss of large choroidal vessels, hyalinisation of the stroma and diffuse choroidal thinning [23].

They are easily distinguished on en-face infra-red OCT images or on OCT sections. Reticular pseudo-drusen (RPD) are not exclusively present in AMD eyes, one study identified that up to 23% of the eyes with no AMD presented RPD compared to 52% with AMD. Within the latter group, RPDs were identified in 49% of eyes with early AMD and in 79% of those with intermediate AMD [24]. This further translates into the observation that the prevalence of RPDs is strongly associated with AMD, as well as with its severity.

Resembling druse, RPD is also a dynamic structure that changes in volume, shape and its relationship with the EZ. 3 different stages have been identified: stage 1. presence of diffuse deposits that contain hyperreflective granular material between RPE and EZ (the former inner segment/outer segment (IS/OS) junction (see Figure 1I); stage 2. mounds of accumulated material that interfere with EZ contour (see Figure 1I); stage 3: disorganization and interruption of EZ (see Figure 1J) [25].

It seems that RPD can reabsorb which would correspond to a stage 4 according to Querques et al.: subretinal deposits disappear through reabsorption or through migration toward internal retinal layers [26].

One study analysed the fellow eye of 200 patients with CNV, 58% of whom had RPD. After a mean follow-up of 2.3 years, 14% developed GA and 36% CNV. Those with RPD developed more often late-stage AMD (61% vs. 33.4%, *p* < 0.001) especially GA (22.4% vs. 2.4% *p* < 0.001), the authors concluding that RPD could be an independent risk factor for GA, but not for CNV (HR 4.93 vs. 1.19) [27]. 

### 4.4. Hyperreflective Foci

Pigmentary visible changes on CFP are due to the presence of HRF on SD-OCT. They appear as well-defined, discrete, high reflectivity lesions in the neurosensitive retina [28], having a similar to or higher reflectivity than the RPE [29] (see Figure 1K).

Leuschen et al. studied 313 patients with AMD and found that 50% presented HRF [4], in line with Schuman et al. that found HRF in 41% of the studied eyes [30]. Their presence seems to be a precursor of larger pigment clumps visible on CFP and represent a progression risk factor for advanced AMD [31]. Furthermore, the association between HRF over the druse and a modified internal refle ctivity seems to indicate an inflammatory process inside the druse and in the outer retina, which will further contribute to AMD progression [32]. The source of these lesions is not completely understood, but it seems that they represent an infiltrate of inflammatory cells such as retinal microglia that induce changes at the level of RPE [33]. Other studies described HRF as activated migrated RPE cells in the internal retinal layers [34], while their capacity to migrate is induced by citokines and other inflammatory mediators as a response to oxidative stress and complement activation [35]. The highest probability for a druse to have HRF is if the central core has low or high internal reflectivity [4]. Another argument for their dynamic evolution is illustrated by the results of a prospective study: after following 300 eyes over a 2-year period, the authors demonstrated that in intermediate AMD, HRF progressively proliferate and migrate towards the internal retinal layers [36]. The AREDS2 ancillary SD-OCT study showed that GA lesions that were identified at 2 years, correlated significantly with the presence and the number of HRF at baseline [28]. Moreover, HRF’s aria correlated significantly in all 3 regions with the progression to advanced AMD: R = 0.610 for HRF 3mm, R = 0.622 for HRF 5mm, and R = 0.614 for HRFTOT (all *p* < 0.001). 

### 4.5. Sub-RPE Hyper-Reflective Columns 

These lesions appear as narrow hyper-reflective columns under the RPE layer and suggest deficiences within it (see Figure 1D) [37]. Moreover, they were observed in 27% of eyes that progressed to nvAMD included in the above mentioned study. Their presence was confirmed 3 months earlier than the onset of GA or exudative changes. The overlying RPE seems intact and the hypothesisbehind these lesions is that some small deficiencies that appear in the RPE layer compromise the integrity of RPE and thus new vessels are easily allowed to invade the subretinal space [37].

### 4.6. Ellipsoid Zone Disruption

One study found out that the decreased absolute and relative reflectivity of EZ prior to the disruption of either RPE or EZ on OCT might indicate early photoreceptor damage [38]. Another study revealed that EZ’s disruption was associated with progression to overall advanced AMD and CNV (odds ratios [ORs]: 17.9 and 30.6; *p* < 0.001), with a similar trend observed for GA [39] (see Figure 1B,C,J).

### 4.7. Drusen with Sub-Retinal Fluid

Subretinal fluid could be detected near a large confluent druse in the adjoining depression in approximately 11% of intermediate AMD [40]. The fluid accumulates only in the concavity between the clustering soft drusen and not on the outward slopes [40] and it will not exceed the adjacent drusen peaks (see Figure 2A). This precise location indicates that mechanical strain is produced by the cluster of coalescent on the outer retinal layers that locally pulls the sensory retina away from its normal position [40]. Moreover, on FA or ICGA no CNV was detected in the analyzed eyes [40].

### 4.8. Acquired Vitelliform Lesion

It represents an accumulation of yellowish subretinal material, characteristic of Best’s vitelliform macular dystrophy and adult foveomacular vitelliform dystrophy. Light and transmission electron microscopy revealed the following structures in the composition of an acquired vitelliform lesion (AVL) acompaning AMD: RPE organelles (3–22% volume), outer segments (2–10%), lipid droplets (0.2–12%) and a flocculent material (57–59%) [41]. Saade et al. performed a review of articles that reported patients with AMD and concomitent AVL and concluded that: most patients had lesions in one eye only, the AVL were localized to the subretinal compartment above the RPE and those treated with anti-VEGF had not significant impact on VA or the AVL aspect [42] (see Figure 2C). Taken together, since HRF and EZ’s disruption are frequently associated when the AVL is at the maximum expansion, leads us to suggest that AVL could be a risk factor for AMD progression.

### 4.9. RPE Thickening

Eyes that progress from intermediate to advanced AMD tend to display thickening of the RPE (*p* = 0.001) [39]. When a separate analysis of the CNV and GA subgroups was performed, eyes that progressed towards CNV had a higher likelihood of RPE thickening compared to GA where it was not significant [39] (see Figure 1B). 

### 4.10. Drusenoid PED

Drusenoid PED was defined as 1/2-disc diameter (DD) of confluent soft drusen under the centre of the macula [43] (see Figure 2B). Roquet et al. followed 61 eyes with treatment naive PED for an average of 4.6 years, of whom 38% had persistent PED, 49% developed atrophy while 13%, choroidal neovascularization. [43] Based on a Kaplan Meier survival analysis, drusenoid PED had a 50% chance of developing GA after 7 years [43]. Drusenoid PED greater than 2 DD associated with metamorphopsia at initial presentation were considered risk factors for progression to atrophy or ingrowth of CNV after 2 years (*p* < 0.01) [43].

### 4.11. Focal Irregular Thinning or the Retina

One study investigated the relationship between photoreceptor layers overlying or adjacent to large druse in eyes with intermediate AMD and found a significant reduction of the photoreceptor outer nuclear layer (ONL) thickness overlying 92% of the drusen [44]. The layer of photoreceptor inner and outer segments was also reduced proportionally with the ONL thickness [44] (see Figure 2D). Curcio et al. showed in a histopathological study that photoreceptors are lost in aAMD, starting with rods in the parafovea, afterwards the RPE becomes dysfunctional, followed by cones’ degeneration [45].

### 4.12. Nascent GA

This entity was first described by Wu et al. in 2014 [46], being defined by several morphological features that precede the onset and development of GA. On SD-OCT examination it appears as a “subsidence” of the OPL and INL with a hypo reflective wedge without a definite loss of photoreceptors or RPE [47]. In 90% of cases, it is found in the central 1.500 µm of the macula [19]. In contrast with established GA, its size is less than 175 µm, the borders are not that sharp as the punched-out borders in GA and the large underlying choroidal vessels are not visible [19]. On OCT scans the absence of ONL, ELM and inner segment ellipsoid zone is noted. One study reported that nascent GA was described with at least 11 months earlier than the onset of GA (range 5–21 months) [46].

GA can develop into different stages, depending on the disappearance of the photoreceptor and RPE layer. According to the Classification of Atrophy Meetings (CAM), GA can be subdivided into: incomplete outer retina atrophy (iORA: thinning of the outer retina with an intact RPE band and no hyper transmission of light into the choroid below Bruch’s membrane (BM)) (see Figure 2D), complete outer retina atrophy (cORA: severe thinning of the outer retina, in the setting of an intact RPE band with intermittent hyper transmission of light) (see Figure 2E), incomplete RPE and outer retinal atrophy (iRORA: degeneration of photoreceptors, an irregular or interrupted RPE band and discontinued hyper transmission of light)(see Figure 2F) and finally complete RPE and outer retinal atrophy (cRORA: degeneration of photoreceptors and a zone of complete disrupted RPE band of at least 250 µm in diameter with the hyper transmission of light) [48] (see Figure 2G).

## 5. Artificial Intelligence’s Contribution in Intermediate AMD Progression

Since OCT analysis of AMD patients provides a large amount of information on the retina, subjective and manual assessment of imaging biomarkers becomes difficult to implement, making it unfeasible in the clinical setting. Therefore, automatic image analysis is needed in order to provide objective and repeatable measurements of quantitative features. 

The advent of improvement in mathematical models, computer graphic processing units and availability of big data has allowed artificial intelligence (AI) using either machine learning (ML) or deep learning (DL) techniques to achieve robust performance in healthcare, especially in Ophthalmology.

Bilc S et al. developed a support tool for segmentation of retina layers, relying on graph theory and geodesic distance. Various gradients such as vertical, horizontal or open-closed were interleaved. The system allows the human expert to intervene after each automatic step in order to validate fine tuning of the automatic segmentation, giving control and transparency on the segmentation process. For AMD patients this method has a limitation: the presence of drusen will not allow properly segmentation of OS-RPE and RPE-CH boundaries [49].

‘Advanced RPE Analysis tool’, a Food and Drug Administration (FDA)-approved software was able to measure drusen volume and RPE’s atrophy by generating maps of sub-RPE drusen and identifiying RPE loss by analyzing hyper-reflectivity in the choroid [50]. Another study managed to automatically segment three retinal boundaries in SD-OCT images of eyes with drusen and GA [51], which was further used in the AREDS2 Ancillary SD-OCT Study to quantify the change in intraretinal HRF distribution. They noted a proliferation and inner retinal migration of HRF during follow-up of intermediate AMD eyes, which were associated with greater incidence of GA at 2 years [52].

Advances in computational image analysis enable researchers to perform more than qualitative or quantitative analysis of AMD OCT imaging features. Quantitative characteristics of a druse such as number, volume, height, reflectivity and longitudinal evolution were used to predict the risk of progressing to nvAMD, with an accuracy of the predictive model of a mean area under the receiver operating characteristic curve (AUC) of 0.74 (95% confidence interval [CI], 0.58, 0.85) [12]. The use of deep learning-based algorithms significantly increased the prediction of AMD progression which further led to the development of AI systems. Some researchers used only imaging biomarkers, while others combined them with genetic and socio-demographic factors.

Some of the late AMD cases developed precisely at the location where a previous druse regressed. This finding motivated researchers to try to predict regression by focusing on the shape, structure, overlying neurosensory layers and short-term longitudinal change at the level of each individual druse. The model predicted drusen events with an AUC performance of 0.75, revealing that the mean druse thickness, maximum druse height and its attenuation had the greatest impact on the regression and progression of intermediate AMD [52]. Regarding HRF, the same authors found that their volume in ONL is related to regression, but not as strongly as druse shape or its attenuation.

Machine learning-based ranking of the morphologic features predicting CNV, or GA conversions highlighted differential features for the separate pathways: for CNV it was either the HRF increase which was mostly associated with druse rank high in predictive value, as well as increase in volume and mean RPE-druse complex (RPEDC), or druse area/volume. The predictive factors for GA were atrophy at the level of RPE+IS/IS segment as well as thinning of the ONL [53]. Another study using a fully automated prediction of GA development from quantitative OCT biomarkers demonstrated that a thinner and a lower reflectivity in RPE and outer photoreceptor segments is an early indicator of regions susceptible to GA growth [54]. The neurosensory alteration identified by the previous studies was correlated with a decrease in cone-mediated sensitivity and rod mediated adaptation time by [55] and also with a mesopic retinal sensitivity and RPE+OS volume [56]. 

Further on, studies tried to find out if there are any specific morphological patterns of OCT in eyes with AMD that develop CNV or GA. One cohort study of 8529 OCT volumes from the HARBOR study identified that eyes progressing to CNV had a substantial druse height at the foveal center and HRF overlying them as well as peaking at 0.5 mm eccentricity contrary to GA that did not exhibit drusen or HRF at the foveal center but instead were found at 0.5 mm eccentricity (i.e., at the edge of the foveal pit) [57]. The difference is further explained by the authors: the lower amounts of drusen in the foveal center could be due to a previous drusen regression that occurred as a first step leading to RPE, photoreceptor and choroidal atrophy.

A more recent study implemented a hybrid sequential prediction model, named Deep sequence, in which longitudinal OCT imaging radiomics and demographic information in a recursive neural network (RNN) model were incorporated to predict the probability of an exudative event in eyes with early and intermediate AMD. The model obtained a very high-performance predicting AMD progression with a 0.96 ± 0.02 AUC, in the short time (within 3 months) and a 0.97 ± 0.02 AUC in the long term (within 21 months) [58].

The study performed by Hallak et al. evaluated factors associated with conversion to CNV, by analysing genetic, demographic and SD-OCT images. The authors revealed that the area occupied by all the individual drusen regions in the OCT topographic map within 3 mm of the foveal centre as well as mean druse reflectivity is potentially associated with conversion. In addition, 1 genetic variant (rs61941274 [*ACAD10* locus]) was associated with conversion to nvAMD [59].

nvAMD typically affects one eye first, then 20% of patients will develop nvAMD in the unaffected eye within 2 years, according to Yim, J. et al. [60]. A different AI system tried to predict whether the fellow eye will convert to nvAMD imminently, defined as in the following 6 months using OCT scans, within a clinically actionable 6-month time window, achieving a per-volumetric-scan sensitivity of 80% at 55% specificity, and 34% sensitivity at 90% specificity. This level of performance corresponds to true positives in 78% and 41% individual eyes, and false positives in 56% and 17% individual eyes, at the high sensitivity and high specificity points, respectively [60].

A pilot study evaluated two deep convolutional neural networks (CNN) in their attempt to predict which eyes would progress to advanced AMD at 2 years: (1) VGG16, a popular CNN for image recognition was fine-tuned, and (2) a novel, simplified CNN architecture was trained from the begining. AMDnet achieved an area under the receiver operating characteristic (ROC) curve (AUC) of 0.89 at the B-scan level and 0.91 for volumes, while for VGG16, with preprocessing AUC was 0.82 for B-scans/0.87 for volumes vs. 0.66 for B-scans/0.69 for volumes without preprocessing [61].

A novel approach used DL based on OCT, CFP, and combination of OCT and CFP. These models consisted of pre-trained VGG-19 and transfer learning using random forest. Following the data augmentation and training process, the DL using OCT alone showed diagnostic efficiency with the AUC of 0.906 (95% confidence interval, 0.891–0.921) and 82.6% (81.0–84.3%) accuracy rate. The DL using CFP alone exhibited AUC of 0.914 (0.900–0.928) and 83.5% (81.8–85.0%) accuracy rate. Combined use of the CFP with OCT increased the diagnostic power: AUC of 0.969 (0.956–0.979) and 90.5% (89.2–91.8%) accuracy rate [62].

In line with previous studies, other inverstigators used the same approach in trying to predict late AMD development. The AUC was similar for the models based on CFP alone (model 1; 0.80), OCT alone (models 2 and 3; 0.82 for both), and when using both methods together (model 4; 0.85). In addition, these models also performed similarly for predicting the end point of atrophic AMD only (AUC, 0.83, 0.84, 0.85, and 0.88 for models 1, 2, 3, and 4, respectively) [63]. An overview of the studies regarding AI is provided in Table 2.

This current review has several limitations. Firstly, it is not a systematic review. In addition, the design of the studies included is heterogeneous. 

## 6. Conclusions

SD-OCT retinal imaging allows to identify biomarkers whose presence or interaction with the surrounding layers predict the conversion from intermediate AMD to advanced AMD, either GA or CNV. Knowledge of these changes is expected to give a deeper understanding of intermediate AMD pathogenesis and could pave the way for finding new treatment options. 

Artificial intelligence could facilitate this process by making it easier to implement, less time consuming, more accurate, since it integrates OCT structural findings with genetic risk and lifestyle. However, the results are still inconsistent due to several confounding factors such as databases, sample sizes or methods. 

## Figures and Tables

**Table 1:** Classification of Age-related macular degeneration.

Stage	Definition
no AMD	No drusen or small drusen ≤ 63 µm, no pigment abnormalities
Early AMD	Medium drusen > 63 µm and ≤125 µm, no pigment abnormalities
Intermediate AMD	Large drusen > 125 µm or any pigment abnormalities
Advanced AMD	nvAMD or GA

AMD = age-related macular degeneration.

**Table 2:** Studies regarding AI and iAMD progression.

First Author	Publication Year	Country	Database	Total Images	Outcome	Performance
Bogunovic [52]	2017	Austria	private dataset	61 eyes/944 drusen	drusen regression	AUC 0.75 (2 years)
Schmidt-Erfurth [53]	2018	Austria	HARBOR	495 eyes	iAMD conversion	AUC 0.68 (CNV) AUC 0.8 (GA)
Russakoff [61]	2019	United States	private dataset	71 eyes	AMD progression	AMDnet: AUC 0.89 B-scan and AUC 0.91 volumes VGG16: AUC 0.82 B-scan and AUC 0.87 volume
Yoo [62]	2019	South Korea	Project Macula	98 eyes	AMD diagnosis	AUC 0.906 (OCT) AUC 0.914 (fundus) AUC 0.969 (mix)
Waldstein [57]	2020	Austria	HARBOR	518 eyes/ 8529 images	progression to CNV/MA	CNV: AUC 0.66 drusen MA: AUC 0.73 HRF
Banerjee [58]	2020	United States	HARBOR	671 eyes/13.954	risk of exudation in iAMD	AUC 0.96 (3 months) AUC 0.97 (21 months)
Yim [60]	2020	UK	private dataset	2795 patients	fellow eye nvAMD prediction	TP in 78% and 41% FP in 56% and 17%

iAMD = intermediate age-related macular degeneration, TP = true positives, FP = false positives, AUC = area under the curve; CFP = colour fundus photography; OCT = optical coherence tomography; CNV = choroidal neovascularization, MA = macular atrophy; GA = geographic atrophy, nvAMD = neovascular age-related macular degeneration.

## Data Availability

Not applicable.

## Supplementary Materials

The following supporting information can be downloaded at: https://www.mdpi.com/article/10.3390/life12030454/s1, Table S1: STARD reporting guidelines score; Table S2. CONSORT-AI reporting guidelines score.
