# Supplementary material for: SD-OCT Biomarkers and the Current Status of Artificial Intelligence in Predicting Progression from Intermediate to Advanced AMD
Source: Life (Basel). 2022 Mar 19;12(3):454. doi: 10.3390/life12030454 (PMC8950761; doi:10.3390/life12030454)
Supplement: Supplementary file 1 [file life-12-00454-s001.zip › life-1612118-supplementary.pdf]

Table S1. STARD reporting guidelines score.

First Author	Country	Year	Journal	STARD Score (% Items Scored)
Yehoshua Z [13]	USA	2011	Ophthalmology	69%
Veerappan M [16]	USA	2016	Ophthalmology	78%
Abdelfattah NS [17]	USA	2016	Investigative Ophthalmology and Visual Science	100%
Schlanitz FG [18]	Austria	2017	British Journal of Ophthalmology	65%
Klein ML [20]	USA	2008	Ophtalmology	56%
Zarubina AV [24]	USA	2016	Ophthalmology	74%
Querques G [26]	France	2012	Investigative Ophthalmology and Visual Science	74%
Finger RP [27]	Australia	2014	Ophthalmology	65%
Christenbury JG [28]	USA	2013	Ophthalmology	78%
Nassisi M [29]	USA	2018	Investigative Ophthalmology and Visual Science	83%
Buch H [31]	Denmark	2005	Acta Ophthalmologica Scandinavica	83 %
Folgar FA [36]	USA	2016	Ophthalmology	83%
Ferrara D [39]	USA	2017	Investigative Ophthalmolology and Visual Science	74%
Roquet W [43]	France	2004	British Journal of Ophthalmology	48%
Sadigh S [44]	USA	2013	Investigative Ophthalmology and Visual Science	57%

Table S2. CONSORT-AI reporting guidelines score.

First Author	Country	Year	Journal	CONSORT-AI Score (% Items Scored)
Bogunovic H [52]	Austria	2017	Investigative Ophthalmology and Visual Science	82%
SChmidt-Erfurth U [53]	Austria	2018	Investigative Ophthalmology and Visual Science	94%
Waldstein SM [57]	Austria	2020	JAMA Ophthalmology	79%
Banerjee I [58]	USA	2020	Sci Rep	85%
Yim J [60]	UK	2020	Nature Med	76%
Russakoff DB [61]	UK	2019	Investigative Ophthalmology and Visual Science	82%
Yoo TK [62]	South Korea	2018	Med Biol Eng Comput	76%
